# Maintenance of *S. aureus* in Co-culture With *P. aeruginosa* While Growing as Biofilms

**DOI:** 10.3389/fmicb.2018.03291

**Published:** 2019-01-09

**Authors:** Paul W. Woods, Zane M. Haynes, Elin G. Mina, Cláudia N. H. Marques

**Affiliations:** ^1^Department of Biological Sciences, Binghamton University, Binghamton, NY, United States; ^2^Binghamton Biofilm Research Center, Binghamton University, Binghamton, NY, United States

**Keywords:** dual species biofilms, *Staphylococcus aureus*, *Pseudomonas aeruginosa*, cystic fibrosis, bronchial epithelial cells

## Abstract

Bacterial biofilms are found in various environmental niches and are mostly comprised by two or more bacterial species. One such example, are the mixed species bacterial biofilms found in chronic lung infections of cystic fibrosis (CF) patients, which include the *Staphylococcus aureus* and *Pseudomonas aeruginosa* bacterial species. *S. aureus* is one of the CF lung initial colonizers and is assumed to be abrogated when *P. aeruginosa* becomes established, eliminating its involvement as the infection evolves. Common models used in research do not mimic the actual progression of the mixed species biofilms thus, in this work we developed an *in vitro* model, where *S. aureus* biofilms establish prior to the introduction of *P. aeruginosa*, simulating a state that is phenotypically more similar to the one found in CF lungs. Overall our results demonstrate that *S. aureus* is not outcompeted, and that timing of inoculation and bacterial concentration affect the final bacterial ratio and quorum sensing related gene expression during the dual species biofilm development.

## Introduction

**FIGURE 1:**
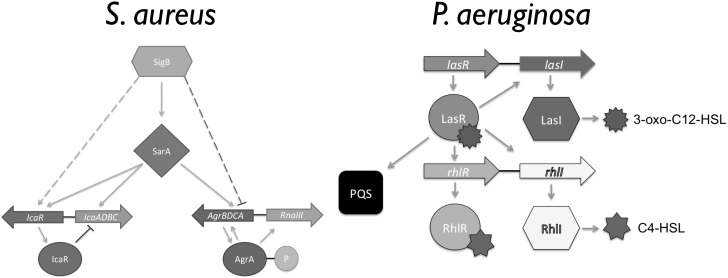
Overview of the *Staphylococcus aureus* and *Pseudomonas aeruginosa* quorum sensing (QS) hierarchy (Adapted from Pearson et al., 1997; Le and Otto, 2015).

Biofilms, bacterial communities with a distinct phenotype compared to free-living planktonic cells, are considered to be responsible for most chronic infections. The biofilm phenotype has several characteristics including: differential expression of efflux pumps, presence of a self-produced extracellular polymeric matrix (EPS) surrounding the cells, an increased resistance to antimicrobials and the ability to escape the host immune response (Stoodley et al., 2002; Southey-Pillig et al., 2005; Hurley et al., 2012; Bjarnsholt et al., 2013; Kostakioti et al., 2013). Thus, biofilm growth results in an increased resistance to environmental stresses and an ability to persist within harsh environments (Stoodley et al., 2002). When gaging their surrounding environment bacteria make use of cell–cell communication, also known as quorum sensing (QS), a necessary survival skill as biofilm infections are normally composed by several bacterial species, such as the ones found in wound infections, eye infections, and lung infections of cystic fibrosis (CF) patients (Recsei et al., 1986; Lyczak et al., 2002; Burmølle et al., 2006; Bjarnsholt et al., 2010b; Stacy et al., 2016). These signaling systems have been shown to regulate EPS, efflux pumps, attachment, and virulence factors known to be associated with disease and bacterial cohabitation (Bjarnsholt et al., 2010a). Gram-negative bacteria make use of acyl-homoserine lactones, while Gram-positive bacteria use short signaling peptides. In *Pseudomonas aeruginosa, las, rhl*, and *pqs* are the main signaling systems (Figure 1; Pearson et al., 1997). In *Staphylococcus aureus*, the *sarA, agrB, and icaR* regulate its ability to survive within a biofilm while in the host, and while coexisting with other bacterial species (Figure 1; Le and Otto, 2015).

In CF patients the bacteria co-existence within the airways leads to lung pathogenesis during infection (Hibbing et al., 2010; Rogers et al., 2010). *S. aureus* is one of the initial microbial colonizers of the CF airway followed by other opportunistic organisms such as *P. aeruginosa and Burkholderia cepacia* (Lyczak et al., 2002; Mashburn et al., 2005; Kahl, 2010). The interaction between *S. aureus* and *P. aeruginosa*, when in co-culture, have been described both *in vivo* and *in vitro*, where bacteria are inoculated simultaneously and at identical concentrations. Most studies suggest that *S. aureus* is predominantly outcompeted by *P. aeruginosa* and thus, has a minimal contribution to the overall course of the infection (Machan et al., 1991; Mashburn et al., 2005; Kahl, 2010; Bragonzi et al., 2012; Filkins et al., 2015). The overall prognosis of a patient is changed once the host’s immune response is activated by the presence of *S. aureus* and *P. aeruginosa*, resulting in alteration of mucus production, neutrophil recruitment, and fluctuations in free metabolites (Machan et al., 1991; Høiby et al., 2011; Alavi et al., 2013; Baldan et al., 2014; Chekabab et al., 2015). In certain instances, the presence of *S. aureus* hampers the host’s immune response to *P. aeruginosa* by inhibiting IL-8, responsible for the recruitment of neutrophils (Joseph et al., 2005; Chekabab et al., 2015). Other studies suggest that when in the presence of *P. aeruginosa, S. aureus* sigma factor B is activated, resulting in a phenotypic change to a small colony variant (SCV) with a decreased metabolic rate (Hoffman et al., 2006; Biswas et al., 2009; Mitchell et al., 2010; Filkins et al., 2015). This *S. aureus* conversion to SCV is commonly associated with an increased resistance to the stress environment found within the lungs of CF patients (Moisan et al., 2006). Common consensus is that *S. aureus* survival in co-culture with *P. aeruginosa* is dependent on its ability to convert to the SCV phenotype (Hoffman et al., 2006). Although a significant progress has been made in co-culturing *S. aureus* and *P. aeruginosa*, the understanding provided by these studies is limited by inferring that *S. aureus* is no longer contributing. Thus, despite the increase in research on multi-species biofilms, their interaction and their interspecies communication is still not completely understood (Recsei et al., 1986; Bjarnsholt et al., 2010a; Stacy et al., 2016).

Driven by this gap in knowledge together with the new findings that 31% of CF patients are co-infected with *P. aeruginosa* and *S. aureus* (Limoli et al., 2016), that older CF patients have higher *S. aureus* infection rates than previously anticipated (Cystic Fibrosis Foundation, 2016), and that *P. aeruginosa* co-isolated with S. *aureus* from CF patients display a lower competitiveness and co-exist with *S. aureus in vitro* (Limoli et al., 2017). Our study focused on determining whether we can simulate the *S. aureus* and *P. aeruginosa* biofilm co-culture found the lung of CF patients.

## Materials and Methods

### Bacterial Strains and Growth Medium

*Staphylococcus aureus* (ATCC 6538) and *P. aeruginosa* PAO1 were used throughout this study. Media consisted of full-strength brain heart infusion medium (BHI) and Lennox LB medium (LB), 20% BHI, and 10% BHI. *P. aeruginosa* quorum sensing mutants used in this study included: *P. aeruginosa* PAO-JP1 (Δ*lasI*), *P. aeruginosa* PAO-JP2 (Δ*lasI/rhlI*), and *P. aeruginosa* PDO100 (Δ*rhlI*) (Pearson et al., 1997; Pesci et al., 1997).

### Growth of Biofilms on Abiotic Surfaces

Single and dual species biofilms of *P. aeruginosa* and *S. aureus* were grown on 2 types of abiotic surfaces: polystyrene wells and silicone tube reactors.

#### Biofilm Growth on Polystyrene Surfaces

Single and dual-species biofilms of *P. aeruginosa* and *S. aureus* were grown on 24 well polystyrene plates, at 37°C with shaking (220 rpm) [MaxQ^TM^ 4000 Benchrtop Orbital Shaker, Thermo Scientific with a 0.75 (19 mm) circular orbit]. An overnight culture of *S. aureus* (400 μL) was used to inoculate the reactors at a concentration of 7 × 10^7^ CFU/mL. Following 1 h of bacterial attachment, the non-attached cells were removed by replenishing the medium (20% BHI medium strength). Medium was subsequently replenished every 12 h for the duration of the experiment. *S. aureus* biofilms developed for a period of 5 days, after which, *P. aeruginosa* was introduced at a ratio of 1 *P. aeruginosa* bacterium to 250 *S. aureus* bacteria. *P. aeruginosa* was allowed to adhere to the pre-existing biofilms and non-colonized surfaces for a period of 1 h, after which the non-attached cells were removed by replenishing the media. Bacterial load within biofilms was assessed at 24 h intervals throughout the experiments. Sampling was attained by removing the supernatant and replenishing the wells with phosphate buffer saline (PBS), after which, biofilms were scraped from the surface using a pipette tip, and the sample was placed onto a microfuge tube, homogenized with a tissue tearor homogenizer, serially diluted, and drop platted onto mannitol salt agar (Difco), *Pseudomonas* isolation agar (Difco), and 50% plate count agar (Difco). Cell viability was determined following 24 h of incubation at 37°C. Controls consisted of axenic cultures of *S. aureus* and *P. aeruginosa*. Additional experiments were performed where biofilm cultures were co-inoculated with *S. aureus* and *P. aeruginosa* at a ratio of 250 to 1.

#### Biofilm Growth on Silicone Tube Reactors

Single and dual-species biofilms of *P. aeruginosa* and *S. aureus* were grown in a silicone tube reactor system, at 22°C under continuous flow conditions, as previously described (Sauer et al., 2002; Davies and Marques, 2009; Marques et al., 2014). An overnight culture of *S. aureus* (1.8 mL) was inoculated at a concentration of 7 × 10^7^ CFU/mL and allowed to attach to the silicone tubing for a period of 1 h under static conditions. The medium (10% BHI) flow was then initiated at (10.8 ml/h) and *S. aureus* biofilms were allowed to establish for a period of 5 days. On day 5, *P. aeruginosa* was introduced at a ratio of 1 *P. aeruginosa* bacterium to 250 *S. aureus* bacteria and dual species biofilms were cultured for further 14 days. During the introduction of *P. aeruginosa* the flow was stopped for a period of 1 h to allow for bacterial attachment. Bacterial viability was monitored at 24 h intervals. Biofilm samples were harvested with the role pin method (Sauer et al., 2002) where the biofilm paste was resuspended in 1 mL of phosphate buffer saline (PBS), homogenized for 20 sec with a tissue tearor homogenizer, serially diluted, and drop platted onto mannitol salt agar (Difco), Pseudomonas isolation agar (Difco), and 50% plate count agar (Difco). Cell viability was determined following 24 h of incubation at 37°C. Controls consisted of axenic cultures of *S. aureus* and *P. aeruginosa*.

### Quantitative Reverse Transcriptase PCR (qRT-PCR)

Relative gene expression was quantified for both *S. aureus* and *P. aeruginosa* biofilms when cultured as single and dual-species. Biofilm samples were collected directly into RNA protect at several time points: 24, 48, 72, 96, and 120 h. RNA was extracted from RNA Protect-treated (Qiagen) samples, using the RNeasy mini kit (Qiagen), with residual DNA degraded using the DNase I amplification grade kit (Invitrogen). A total of 0.5 μg of RNA was used for cDNA synthesis, and cDNA was generated using a RETROscript^®^ Kit (Ambion). Quantitative reverse transcriptase PCR (qRT-PCR) was performed with an Eppendorf Mastercycler ep realplex instrument (Eppendorf AG, Hamburg, Germany) and the KAPA SYBR FAST qPCR Kit (Kapa Biosystems, Woburn, MA, United States) with the oligonucleotides (obtained from Integrated DNA Technologies, Coralville, IA, United States) listed in Table 1. No reverse transcriptase (NRT) reactions and no template control (NTC) were performed to ensure the absence of foreign or genomic DNA contamination during the preparation of samples and master mix. Relative transcript quantitation was accomplished using the ep realplex software (Eppendorf AG) with the transcript abundance (based on the threshold cycle value Ct) of *S. aureus* normalized to *tpiA* (control), and *P. aeruginosa* normalized to *mreB* (control), before the determination of transcript abundance ratios. Verification of single-product amplification was carried out through the analysis of the melting curves.

**Table 1:** Primers used in this study.

Gene	Forward primer	Reverse primer	Amplicon size (bp)	Melting temperature (°C)
*tpiA/SA*	AGATAATGGTGCGTTCACAG	TGTTTGAAAATAGCGTGCGC	150	FW: 56.4, RV: 56.4
*agrB/SA*	GCAAGTACGTTTAGGGATGC	CGAAGACTTTGCATGAGCAC	163	FW: 58, RV: 58
*sarA/SA*	CAGCGAAAACAAAGAGAAAG	TCGTTGTTTGCTTCAGTGAT	233	FW: 54, RV: 54
*sigB/SA*	GGTCATCTTGTTGCCCCATA	GCCGTTCTCTGAAGTCGTGA	149	FW: 51.8, RV: 53.8
*icaR/SA*	CCGTCATACCCCTTCTCTGA	CCAGAAAATTCCTCAGGCGT	244	FW: 51.8, RV: 53.8
*mreB/PA*	CTTCATCAACAAGGTCCACGA	GCTCTTCGATCAGGAACACC	152	FW: 59.5, RV: 60.5
*lasI/PA*	CAGGTTTCCGGTTCGTGG	TTCCTTGCCGTGCAGAAG	87	FW: 56.3, RV: 56.3
*lasR/PA*	AGCTGGAACGCTCAAGTG	TGGCTGTCCTTAGGCAAC	109	FW: 56.3, RV: 56.3
*rhlR/PA*	GACCAGCAGAACATCTCC	CTGGGTCAGCAACTCGAT	78	FW: 56.3, RV: 56.3
*rhlI/PA*	GTTCGACCATCCGCAAAC	ACGTCCTTGAGCAGGTAG	105	FW: 56.3, RV: 56.3
*pqsH/PA*	GAGACGCTGATCCTGTTC	CGATTCCCACTGACCAAG	96	FW: 56.3, RV: 56.3


### Effect of *P. aeruginosa* Culture Supernatant on *S. aureus*

Inhibition of *S. aureus* growth by compounds present in the supernatant of *P. aeruginosa* cultures was evaluated with late stationary phase cultures (24 h) of *P. aeruginosa* WT, PAO-JP1 (Δ*lasI*), PAO-JP2 (Δ*lasIrhlI*), and PDO100 (Δ*rhlI*). Cultures were grown 37°C with agitation (220 rpm) in BHI medium. Culture biomass was standardized to 10^8^ CFU/mL, after which, 5 mL of each culture were sedimented by centrifugation at 16,000 × g for 10 min at 4°C. The supernatant was removed and filtered through a 0.2 μm syringe filter. Spent medium was stored at -80°C until further use. An overnight culture of *S. aureus* was standardized to 0.5 McFarland, and a lawn was prepared on Mueller Hinton Agar with an agar concentration of 1.5%. Wells with 0.5 cm diameter were punctured into the agar, agar plugs were removed, discarded, and 150 μL of supernatant were added to each well. Controls consisted of sterile medium processed similarly to bacterial cultures. Cultures were then incubated at 37°C for a period of 24 h. Zones of inhibition were measured at 12 and 24 h.

## Results

### Pre-established *S. aureus* Biofilms Are Not Completely Outcompeted by *P. aeruginosa*, When Cultured on Abiotic Surfaces

**FIGURE 2:**
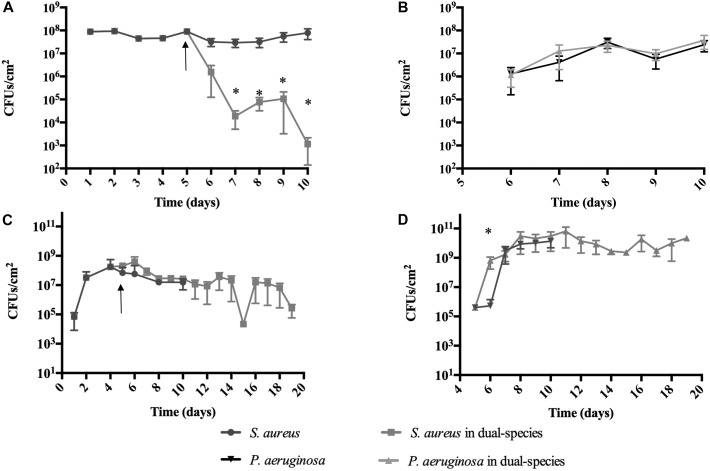
*Staphylococcus aureus* persists in co-culture with *P. aeruginosa* on abiotic surfaces. *S. aureus* was allowed to establish a biofilm for 5 days prior to the introduction of *P. aeruginosa. P. aeruginosa* was introduced at a concentration of 1 *P. aeruginosa* to 250 *S. aureus*. Dual-species growth and bacterial maintenance was determined in various growth conditions: **(A,B)** 20% BHI at 37°C under agitation at 220 rpm, **(C,D)** 10% BHI at 22°C under continuous flow conditions. Controls consisted of biofilms of axenic cultures. Bacterial viability and strain ratios were evaluated daily. **(A)** *S. aureus* viable counts, **(B)** *P. aeruginosa* viable counts, **(C)** *S. aureus* viable counts, **(D)** *P. aeruginosa* viable counts. ^∗^*p* < 0.01 comparing to the axenic cultures. Arrow indicates introduction of *P. aeruginosa*.

*S. aureus* and *P. aeruginosa* are commonly co-isolated from chronic wounds, catheter infections, eye infections, skin infections, and lung infection in CF patients (Machan et al., 1991; Hoffman et al., 2006; Baldan et al., 2014). However, most previous attempts to establish and study these 2 microorganisms as dual-species biofilms under laboratory conditions has been futile as, *S. aureus* is mainly outcompeted and eradicated (Baldan et al., 2014; Filkins et al., 2015). Thus, in this work, we approached culturing these dual-species biofilms by attempting to simulate *in vivo* conditions, where *S. aureus* biofilms are first established followed by the introduction of *P. aeruginosa*. To achieve this, *P. aeruginosa* was inoculated into pre-cultured (5 day old) *S. aureus* biofilms grown in 20% BHI medium, either at room temperature (RT) or 37°C (Figure 2), at the ratio of 1 *P. aeruginosa* to 250 *S. aureus.*

When cultured at 37°C in 24-well plates (Figures 2A,B and Table 2), 24 h following its introduction, *P. aeruginosa* made up 50% of the overall bacterial cells within the dual-species biofilms, where a 2-Log reduction of the *S. aureus* viable cells occurred (Figure 2A). The reduction of *S. aureus* viable cells continued up to 48 h, where it reached 5 × 10^3^ CFU/cm^2^ (0.3% of the total population of cells) after which, increased slightly, followed by a bacterial reduction to day 2 levels, by day 5 (Figure 2A). When culturing the biofilms at RT (Figures 2C,D and Table 3) the introduction of *P. aeruginosa*, led to a minimal reduction of *S. aureus* viable cells, with the exception of day 10, where a significant decrease of *S. aureus* occurred, before recovering up to day 14, where a second decrease in *S. aureus* viability occurred (Figure 2C). These results could indicate that, in both culture conditions, the dual-species reached a co-relationship stability.

**Table 2:** Ratio of *Staphylococcus aureus* and *Pseudomonas aeruginosa* when co-cultured in dual-species biofilms on 24-well plates at 37°C with agitation.

Day	0	1	2	3	4	5
*S. aureus*	100%	56.4%	0.3%	0.2%	2%	0.001%
*P. aeruginosa*	0%	43.6%	99.7%	99.8%	98%	99.9%


*P. aeruginosa was introduced at day 5.*

**Table 3:** Ratio of *S. aureus* and *P. aeruginosa* when co-cultured in dual-species biofilms on tube reactors at 22°C.

Day	0	1	2	3	4	5	6	7	8	9	10	11	12	13	14
*S. aureus*	99.97%	88.8%	4.6%	0.05%	0.05%	0.04%	0.02%	0.06%	0.2%	1.1%	0.001%	0.09%	0.5%	0.1%	0.001%
*P. aeruginosa*	0.03%	11.2%	95.4%	99.95%	99.95%	99.96%	99.98%	99.94%	99.8%	98.9%	99.99%	99.91%	99.5%	99.9%	99.99%


*P. aeruginosa was introduced at day 5.*

When in co-culture with *S. aureus*, the cell viability of *P. aeruginosa* remained constant from day 1 of co-culture onward (Figures 2B,D). However, the total bacterial composition of the dual species biofilms changed throughout the experiment and the percentage of *P. aeruginosa* increased overtime. At day 1 of co-culture, *P. aeruginosa* represented ≤50% of the overall bacterial population, this percentage increased to ≥98% of the total population by day 10 (Tables 2, 3).

Comparing to single species growth, the introduction of *P. aeruginosa* led to a significant decrease of *S. aureus* when co-cultured in 24 well plates (Figure 2A and Table 2). This decrease was not observed within tube reactors (Figure 2C) for most of the experimental duration. *P. aeruginosa* cell viability was identical in the presence or the absence of *S. aureus*, with the exception of the tube reactor cultures where at day 1, the cell viability was significantly higher when co-cultured with *S. aureus* (Figure 2D and Table 2).

### Co-inoculation of *S. aureus* and *P. aeruginosa* Biofilms Does Not Eradicate *S. aureus*

Previous research has demonstrated that when co-inoculating *S. aureus* and *P. aeruginosa* at a 1:1 ratio, *P. aeruginosa* completely outcompetes *S. aureus* (Baldan et al., 2014; Filkins et al., 2015). Upon finding that the introduction of *P. aeruginosa* into pre-established *S. aureus* biofilms led to dual-species biofilms that reached a co-relationship equilibrium and not to an eradication of *S. aureus* (Figure 2), we decided to evaluate whether the out-competition would still occur at the 250:1 ratio of *S. aureus*:*P. aeruginosa*.

**FIGURE 3:**
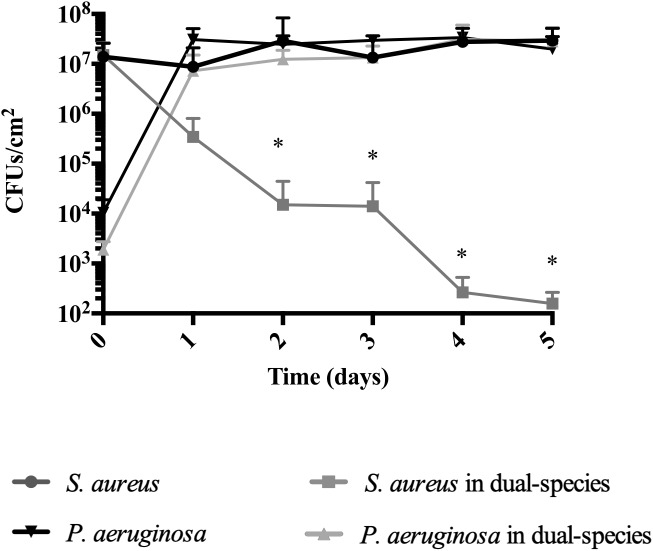
*Staphylococcus aureus* persists in dual-species biofilms when co-inoculated with *P. aeruginosa*. Dual-species biofilms were initiated on a polystyrene surface with *S. aureus* and *P. aeruginosa* at a concentration of 250:1. Biofilm growth was monitored for a period of 5 days under several conditions: 20% BHI at 37°C under agitation at 220 rpm. Media was replaced at 12 h intervals and bacterial viability and strain ratios were evaluated daily. Controls consisted of biofilms of axenic cultures. Bacterial viability and strain ratios were evaluated daily. ^∗^*p* < 0.001 comparing to the axenic culture.

At day 5 of dual-species cultures on abiotic surfaces, when using BHI media at 37°C, we found that *S. aureus* was not eradicated, as anticipated (Figure 3 and Table 4). At day 0, following 1-h inoculation, cells attached to the wells consisted of 99.99% *S. aureus* (10^7^ CFU/cm^2^), and 0.01% *P. aeruginosa* (10^3^ CFU/cm^2^). This ratio was inverted by day 2, where the bacterial biomass consisted of 4.5% *S. aureus* (5 × 10^5^ CFU/cm^2^) and 95.5% *P. aeruginosa* (2 × 10^7^ CFU/cm^2^). Throughout the remainder of the experiment, *S. aureus* decreased at a steady rate, reaching 10^2^ CFU/cm^2^ (0.0005%) by day 5 (Figure 3). While in co-culture, the *P. aeruginosa* cell viability remained constant from day 1 onward, a pattern identical to the one observed for *P. aeruginosa* axenic cultures. *S. aureus* axenic cultures also remained constant throughout the experiments (Figure 3).

**Table 4:** Ratio of *S. aureus* and *P. aeruginosa* when co-cultured in dual-species biofilms on 24-well plates at 37°C with agitation.

Day	0	1	2	3	4	5
*S. aureus*	99.9%	4.5%	0.1%	0.1%	0.0008%	0.0005%
*P. aeruginosa*	0.01%	95.5%	99.9%	99.9%	99.9%	99.9%


*P. aeruginosa and *S. aureus* were co-inoculated at a ratio of 1:250.*

Thus, under the conditions used in this study, independently of being inoculated simultaneously or staggered, *S. aureus* is not eradicated when in dual-species biofilms, as long as *P. aeruginosa* is initially present at a significantly lower concentration than *S. aureus*. This provided us with means to further study their interactions.

### Quorum Sensing (QS) Expression in Dual Species Biofilms

**FIGURE 4:**
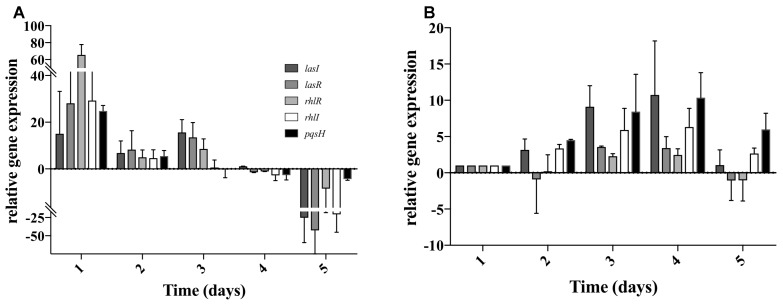
*Pseudomonas aeruginosa* QS gene expression when co-cultured with S. aureus on tube reactors. *S. aureus* biofilms were cultured in tube reactors for a period of 5 days previous to the introduction of *P. aeruginosa*. Once *P. aeruginosa aeruginosa* was introduced (day 0), samples were taken at 24 h intervals for a period of 120 h. **(A)** relative expression of *P. aeruginosa* compared to single species biofilms, **(B)** relative expression of *P. aeruginosa* QS genes in dual-species biofilms compared to day 1 of co-culture. Changes of relative expression were considered significant when a twofold change was present.

**FIGURE 5:**
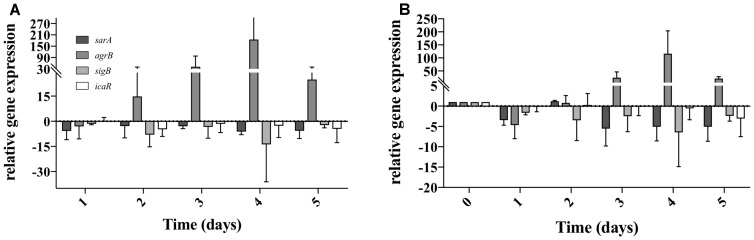
*Staphylococcus aureus* QS gene expression when co-cultured with *P. aeruginosa* on tube reactors. *S. aureus* biofilms were cultured in tube reactors for a period of 5 days previous to the introduction of *P. aeruginosa*. Once *P. aeruginosa aeruginosa* was introduced (day 0), samples were taken at 24 h intervals for a period of 120 h. **(A)** relative expression of *S. aureus* QS genes in dual-species biofilms compared to single species biofilms. **(B)** Relative expression of *S. aureus* single species biofilms compared to time 0. Changes of relative expression were considered significant when a twofold change was present.

As *S. aureus* was not completely eradicated when in co-culture with *P. aeruginosa* (Figure 2), we quantified the relative gene expression of QS related genes throughout the 5 days of co-culture. This was performed in experiments when *S. aureus* was allowed to establish for a period of 5 days previous to the introduction of *P. aeruginosa*. In *P. aeruginosa*, all the QS genes chosen were up regulated once introduced into the preexisting *S. aureus* biofilms, comparing to single species biofilms at identical culture time (Figure 4A). By day 3 *rhlI* and *pqsH* were down-regulated and by day 4, all QS genes presented no change or were down regulated compared to single species biofilms (Figure 4A). When comparing to day 1 of dual-species (Figure 4B), *P. aeruginosa rhlR* and *lasR* presented no significant relative gene expression change at days 2 and 5, being slightly up-regulated at days 3 and 4. Relative expression of *rhlI, lasI*, and *pqsH* was up regulated throughout the experiment (Figure 4B). When quantifying the relative expression of *S. aureus* QS related genes, *agrB* was upregulated from day 2 onward, while *sarA, sigB*, and *icaR*, were slightly downregulated, when compared to *S. aureus* growing alone (Figure 5A). When comparing to day 0 of co-culture, *agrB* was upregulated from day 3, while no significant change was observed for *sigB* and *icaR*, and *sarA* was slightly downregulated (Figure 5B).

### *S. aureus* Thrives When *P. aeruginosa* Quorum Sensing Is Inactivated

**FIGURE 6:**
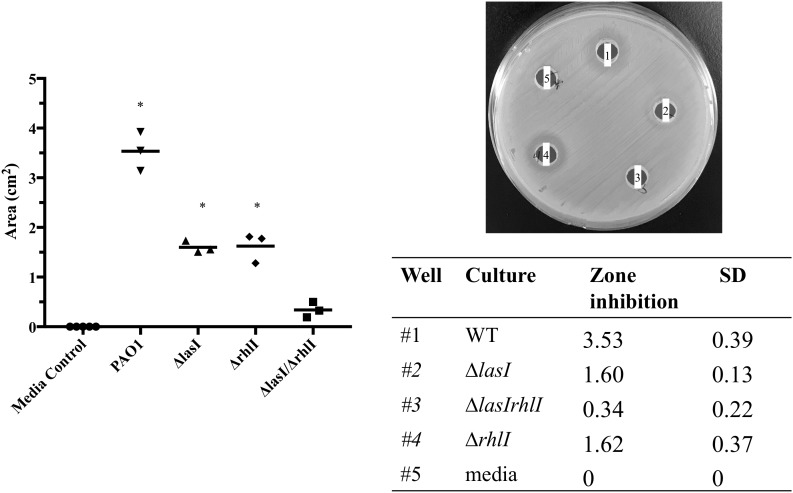
Effect of *P. aeruginosa* mutant supernatant on *S. aureus* growth. Supernatant of the PAO1 WT, Δ*lasI*, Δ*lasIrhl*, and Δ*rhll* strains of *P. aeruginosa* were added to an agar plate containing a lawn of *S. aureus*. The lawns were allowed to grow for 24 h and zones of inhibition were measured using ImageJ. ^∗^p < 0.001 compared to media control.

**FIGURE 7:**
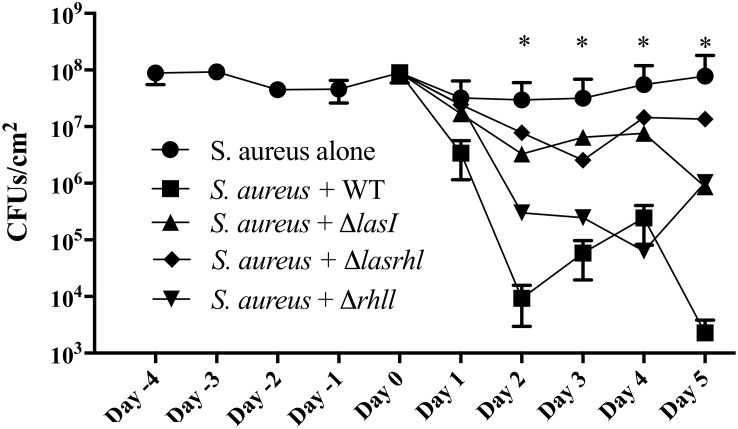
Effect of quorum sensing inactivation on the presence of *S. aureus* when in co-culture with *P. aeruginosa*. Biofilms of *S. aureus* were allowed to establish for a period of 5 days in microtiter plates after which, *P. aeruginosa* was introduced at a 1:250 ratio. *P. aeruginosa* strains consisted of PAO1 WT, Δ*lasI*, Δ*lasIrhl*, and Δ*rhll*. Upon introduction of *P. aeruginosa*, viability of dual-species biofilms was quantified for further 5 days. Biofilms were cultured in 10% BHI at 37°C under static conditions. Media was exchanged every 12 h. ^∗^*p* < 0.001 all strains compared to *S. aureus* alone.

Considering the up-regulation of the relative expression of the *rhl* and *las* genes during the first 2 days (Figure 4), we then evaluated the effect of their absence on *S. aureus* growth. We found that the supernatant of Δ*lasI* and Δ*rhlI* inhibited *S. aureus* growth albeit at a lower rate than WT (Figure 6). Δ*lasIrhlI* did not inhibit *S. aureus* growth. Furthermore, co-cultures of *P. aeruginosa* WT and *S. aureus* led to a 4 Log reduction of the latter by day 2, with a slight cell recovery by day 4, which decrease again to a 4 Log reduction by day 5. In contrast, mutation of QS related genes in *P. aeruginosa* led to a less effective removal of *S. aureus* (Figure 7). Inactivation of *lasI* as well as both *lasI* and *rhlI* genes (Δ*lasIrhlI*) led to a 1 Log reduction of *S. aureus* viable cells by day 2 of co-culture (Figure 7). Inactivation of *rhlI* led to an initial 2.5 Log reduction of *S. aureus* followed by a recovery to levels found when in co-culture with other QS mutants (Figure 7). Overall, the relation of *P. aeruginosa* with *S. aureus* viability decrease was WT > Δ*rhlI* > Δ*lasI = ΔlasIrhlI.* In contrast, the presence of *S. aureus* did not have an effect on *P. aeruginosa* WT (Supplementary Figure 1) but, resulted in a decreased of the cell number of all QS mutant in the first 3 days of co-culture (Supplementary Figure 1).

## Discussion

Natural occurring biofilms are comprised of multiple species of bacteria coexisting in a single environment, however the interactions between the different bacterial species and the role of each species within these multi-species biofilms is mostly unknown. Thus, it is important to study these interactions in conditions that mimic *in vivo* conditions. In wounds, as well as in the lungs of CF patients, it is common to co-isolate *S. aureus* and *P. aeruginosa*, although it has been challenging to co-culture them in the laboratory. Our study focused on the ability of *S. aureus* to persist in dual-species biofilms when co-cultured with *P. aeruginosa*, both on abiotic surfaces and bronchial epithelial cells. Ordinarily, studies involving two or more bacterial species growing as biofilms initiate cultures where bacteria are inoculated simultaneously and at identical concentrations.

Here we attempted to mimic *in vivo* situations, where *S. aureus* infections are established previous to *P. aeruginosa.* When using this approach, we found that in most cases, *S. aureus* was able to maintain a substantial concentration within the dual species biofilms, albeit with a decrease presence with the increase of time of coexistence with *P. aeruginosa* (Figures 1, 2). *P. aeruginosa* however, was not affected by the presence of *S. aureus* (Figures 1, 2). In all of the biofilm experiments involving tube reactors it was observed that viability of *S. aureus* within the dual-species biofilms fluctuated, showing a somewhat cyclical pattern (Figure 1). When *S. aureus* was allowed to reach the stage of a mature biofilm prior to the introduction of *P. aeruginosa*, it persisted within the dual species biofilm at a higher concentration compared to co-inoculation (Figure 2), suggesting that established biofilms of *S. aureus* have a greater resistance to removal by *P. aeruginosa*.

*Pseudomonas aeruginosa* produces multiple virulence factors that contribute to the removal of *S. aureus* from dual species cultures, with the *las* and *rhl* system being the major regulators of these factors (Wu et al., 2001; Bjarnsholt et al., 2010a). In this study we quantified the relative expression of several *P. aeruginosa* QS genes during biofilm maturation when cultured alone and in co-culture with *S. aureus*. Overall, we found that when in dual-species the majority of *P. aeruginosa* QS genes were up-regulated within the initial 3 days decreasing from that point onward, compared to single species (Figure 3), similarly to when comparing to day 1 of co-culture (Figure 3). These findings suggest that an equilibrium is reached between the bacterial species allowing for co-existence. The effect of QS on the co-cultures was further confirmed by the decreased removal of *S. aureus* (Figure 6) when co-cultured with mutants lacking one or both major QS synthases (*lasI, rhlI*, and *lasI/rhlI*), confirming previous findings (Ciofu et al., 2010; Alavi et al., 2013). In addition, exposure of *S. aureus* to the supernatant of each of the *P. aeruginosa* mutants resulted in a decreased zone of inhibition compared to the wild type, with the lowest inhibition occurring in the absence of both synthases (Figure 5).

When determining the relative gene expression of several of the major genes involved in *S. aureus* biofilm formation we found that they were mostly down-regulated compared to single species, with the exception of *agrB* (Figure 4), which is one of the major components of the *agr* auto inducing peptide signaling system in *S. aureus* (Recsei et al., 1986; Boles and Horswill, 2008). The increase in expression *agrB* coincided with the rebound of *S. aureus* viability found during the viable counts, and the stabilization of QS gene expression in *P. aeruginosa* (Figures 1A, 3, 4). These genes are commonly associated with virulence in *S. aureus*, and this increase in expression could suggest a phenotypic change leading to the increased presence of *S. aureus* on day 4 of dual-species culture (Goerke and Wolz, 2004).

Overall, these data suggest that quorum sensing plays an essential role in the co-existence of these two bacterial species, and that our growth model enables the study and the achievement of further insight into their interactions throughout the biofilm development.

The model used throughout this study demonstrates the ability of *S. aureus* to persist within a dual-species biofilm that contains both *S. aureus* and *P. aeruginosa.* Mimicking the patterns found within an actual infection enabled the co-culture of these two species and the further understanding of their interactions, during the dual-species biofilm development. Further studies using this model will allow for a better understanding of the interactions between *S. aureus* and *P. aeruginosa* and how they co-exist in the environment and contribute to infection and chronic diseases.

## Author Contributions

CM conceived the concept. PW, ZH, and EM carried out the experiments. CM and PW co-wrote the paper. All authors discussed the results and comments on the manuscript.

## Conflict of Interest Statement

The authors declare that the research was conducted in the absence of any commercial or financial relationships that could be construed as a potential conflict of interest.
